# A Randomized Controlled Pilot Study of the Triple Stimulation Technique in the Assessment of Electroacupuncture for Motor Function Recovery in Patients with Acute Ischemic Stroke

**DOI:** 10.1155/2013/431986

**Published:** 2013-06-10

**Authors:** Feng Tan, Xuewen Wang, Hui-qin Li, Lin Lu, Ming Li, Ji-huang Li, Meifeng Fang, Di Meng, Guo-qing Zheng

**Affiliations:** ^1^Department of Neurology, Foshan Hospital of TCM, Affiliated Hospital of Guangzhou University of Chinese Medicine, Foshan 528000, China; ^2^The Center of Neurology and Rehabilitation, The Second Affiliated Hospital of Wenzhou Medical College, Wenzhou 325027, China

## Abstract

The objective of this pilot study was to objectively assess electroacupuncture for motor function recovery in patients with acute ischemic stroke using the triple-stimulation technique (TST). The patients received either electroacupuncture plus western conventional medication (WCM) (*n* = 32) or single WCM (*n* = 31) for 14 days. The total clinical effective rate was statistically significantly superior in electroacupuncture group to that in WCM group (*P* < 0.01). Fugl-Meyer Assessment Scale (FMA) score, National Institutes of Health Stroke Scale (NIHSS) score, and TST_ratio_ were statistically more significant in electroacupuncture group than those in WCM group (*P* < 0.01). There was positive correlation between TST_ratio_ and NIHS score both before and after treatment (*P* < 0.01) and negative correlation between TST_ratio_ and FAM score both before treatment and after treatment (*P* < 0.01). Comparing between the two groups or between pretreatment and posttreatment, adverse events, electrocardiogram, liver function, and kidney function showed no statistically significant difference (*P* > 0.05). In conclusion, electroacupuncture was beneficial for the motor function recovery of patients with acute ischemic stroke and was generally safe. TST can be used for quantitative evaluation of electroacupuncture for motor function recovery in patients with acute ischemic stroke because it can objectively analyze the injury and recovery of corticospinal tract impairments.

## 1. Introduction

Stroke is the second most common cause of death after cancer worldwide, and low-income countries are the most affected by the high rates of stroke mortality and burden [1]. Particularly, stroke has become the leading cause of death among all diseases in China, which has one-fifth of the total population (1.3 billion) in the world. However, the only Food and Drug Administration-approved therapy for acute ischemic stroke (AIS) remains the thrombolytic agent recombinant tissue-type plasminogen activator (rtPA) within the limited 4.5-hour timeframe [2]. In addition, symptomatic intracranial hemorrhage (ICH) is a devastating complication of intravenous thrombolysis treatment and is associated with high mortality [3]. Therefore, complementary and alternative medicine (CAM) therapies are increasingly used in patients with stroke adjunct to conventional treatment. Acupuncture, a form of CAM, is one of the oldest medical modality in the world which has played an important role in the medical care of stroke patients for more than 3000 years [4]. In the past decades, a number of randomized controlled trials were conducted to address efficacy and safety of acupuncture for improving motor function recovery of patients suffering from ischemic stroke, but the results are not conclusive [5]. 

Motor impairment is a frequent complication after stroke. The ability to live independently after stroke depends largely on the reduction of motor impairment and the recovery of motor function [6]. Advances in neurophysiological assessments with transcranial magnetic stimulation (TMS) have provided new ways to measure the extent of stroke damage and understand the anatomical and functional changes in the motor system at given time points during the course of recovery [7]. Recently, a novel TMS technique involving two collisions, the triple-stimulation technique (TST), links the central to peripheral conduction and suppresses desynchronization of the motor evoked potentials (MEPs), and this technique enabled a quantitative electrophysiological measurement of the central motor-conduction failure that causes the patient's disability [8]. The TST was reported to be 1.5~2.75 times more sensitive than conventional TMS for detecting corticospinal conduction failure [9–11]. Nowadays, TST has been applied for various disorders to quantify assessment of the central conduction failure, suggesting that TST was useful in quantifying the benefits of treatments in disorders such as multiple sclerosis, amyotrophic lateral sclerosis, and spondylotic myelopathies, or cerebral plasticity in the course of rehabilitation programmes in disorders such as stroke [10]. Thus, TST is a useful diagnostic tool and provides an accurate objective quantification in follow-up studies, including assessment of the efficiency of treatments. Furthermore, the most important period of recovery is at the acute and subacute stages during the clinical course of ischemic stroke [12]. In the present study, we thus conducted a randomized controlled pilot study using the TST to objectively evaluate electroacupuncture (EA) for motor function recovery in patients with the first-ever ischemic stroke and within the first three days of stroke onset.

## 2. Methods

This clinical study was designed as a randomized, controlled trial and was conducted between June 2010 and March 2011 in China. The trial used the two-group parallel design where 63 cases of AIS patients were randomized in a 1 : 1 ratio to receive EA plus western conventional medication (WCM) or single WCM treatment for a total of 14 days. The efficacy and safety of EA was mainly assessed by using TST and several neurological outcome scales after 14-day treatment. The study was conducted in accordance with the World Medical Association Declaration of Helsinki and China's regulations and guidelines on good clinical practice. Ethical approval for the trial was obtained from the Ethics Committee of the local hospital Ethical Review Board. Written informed consent was obtained from all subjects.

### 2.1. Participants

Subjects were considered eligible to be enrolled in the study only when all of the following inclusion criteria were met: (1) a diagnosis of AIS according to the Chinese national criteria in *Diagnostic Essentials of Various Cerebrovascular Diseases* revised at the Fourth National Conference of China Society of Medicine on Cerebrovascular Diseases in 1995 [13]. The diagnosis of AIS must be confirmed by both CT and MRI; (2) within in 72 hours from the onset of stroke; (3) motor deficits in at least one limb, and National Institutes of Health Stroke Scale (NIHSS) score that is greater than or equals to 4; (4) the age ranged from 35 to 80 years; (5) all subjects must participate of their own free will and sign an informed consent form. 

The following exclusion criteria were applied: (1) contraindication of TMS; (2) suffering from neural or musculoskeletal disease that affects function recovery before AIS; (3) more than 72 hours after the onset of stroke; (4) patients suffering from transient ischemic attack, subarachnoid hemorrhage, ICH, or cerebellar infarction; (5) patients who presented with conscious disturbance, aphasia, and dementia after stroke onset; (6) emergency and critical patients who were not suitable for acupuncture such as acute myocardial infarction, serious infection, active tuberculosis, hepatic failure and/or renal failure, and upper gastrointestinal bleeding; (7) patients age < 35 years old or >80 years old; (8) those who refuse to sign an informed consent form.

The terminal criteria were as follows: (1) those who did not meet the inclusion criteria but were included in the trial; (2) patients who did not obey the trial plan to take medication and affected the efficacy evaluation; (3) patients who withdraw from the trial if any of the serious adverse events happened; (4) patients whose condition deteriorated or when serious complications occurred; (5) patients who stop participating due to one's own free will during the trial. Patients could withdraw from the study at any time and for any reason.

### 2.2. Participant Flow, Baseline Evaluation, and Randomization

**Figure 1:**
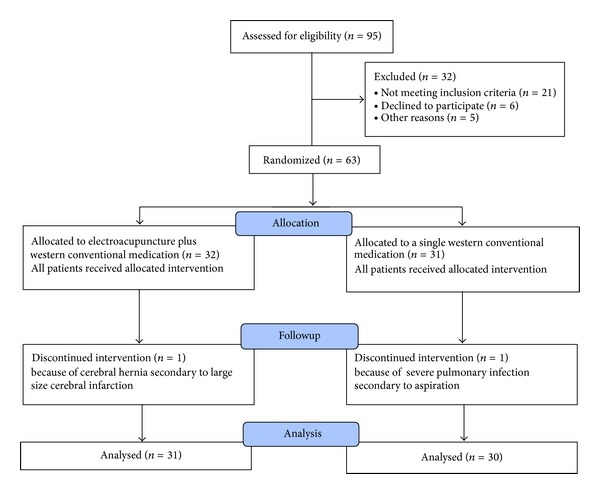
Participant flow diagram.

Based on inclusion criteria, 95 patients were screened and 32 patients were excluded due to not meeting inclusion criteria, declining to participate, and/or other reasons. After the patients have fully understood the study and have signed the informed consent, the baseline evaluation was recorded and assessed by a responsible doctor, including age, sex, height, body weight, history, medication history, drinking/smoking history, risk factors (history of hypertension, stroke, diabetes, and cardiovascular disease), and type of limb paresis. NIHSS score was carried out by a trained doctor. Basic life signs such as breathing, heart rate, blood pressure, and pulse were recorded. The lesion site and volume of cerebral infarction were also assessed by a CT and MRI scan. The infarct volume was divided into 3 types according to the Pullicino formula (length × width × number of CT or MRT scanning positive layer/2). They are large size infarct (>10 cm³), medium size infarct (5~10 cm³), and small size infarct (<5 cm³). All of the patients should take lab tests of liver and kidney function. The qualified subjects finished TST_test_ level and Fugl-Meyer Assessment Scale (FMA) score. Sixty-three AIS patients signed informed consent, finished baseline evaluation, and entered random process. These patients were randomly divided into EA plus WCM treatment group (EA group, *n* = 32) and WCM control group (WCM group, *n* = 31) by using a random number table. The random numbers were sealed in opaque envelopes and the researcher opened the envelope only to start the intervention. During the trial, 2 patients were transferred to intensive care unit from general ward and discontinued the intervention because of one cerebral hernia secondary to large size cerebral infarction and the other severe pulmonary infection secondary to aspirations. Ultimately, statistical analyses were conducted on the results from 31 patients of EA treatment group and 30 patients of WCM control group (Figure 1, participant flow diagram).

### 2.3. Interventions

Based on the Standards for Reporting Interventions in Clinical Trials of Acupuncture (STRICTA) 2010 checklist, we reported interventions in present clinical trial of electroacupuncture as follows: (1) acupuncture rationale: the oldest and greatest extant classic TCM literature Huangdi Neijing (*Huangdi's Internal Classic*) recorded *Wei* Syndrome (flaccidity syndrome). Under this book's Suwen (*Plain Questions*) section, chapter 44 *Wei* Syndrome described that the main acupoints for the treatment of *Wei* syndrome should be specifically selected from the Yangming Meridian in the following manner: “When treating *Wei* syndrome, doctor should specifically target Yangming Meridian,… as Yangming is the source of nourishing for all the *Zang-Fu *internal viscera, only with this nourishment can the tendons, bones, and joints be lubricated.” Therefore, we mainly selected the acupoints of Yangming meridians because they are full of qi and blood whose unblocked circulation is beneficial to recover the affected limbs including paralysis of stroke. (2) Details of needling: acupoints were mainly selected in large intestine meridian/channel of hand yang brightness (LI) and stomach meridian/channel of foot yang brightness (ST) located on the subjects' hemiparetic limb. They are upper limb: Jianyu (LI 15) plus Jianliao (TE 14), Quchi (LI 11) plus Hegu (LI 4), and Chize (LU 5) plus Neiguan (PC 6) and lower limb: Zusanli (ST 36) plus Yanglingquan (GB 34), Fenglong (ST 40) plus Xuanzhong (GB 39), and Sanyinjiao (SP 6) plus Taichung (LR 3). The Huanqiu brand of sterile needles, 1.5 inch in length (0.25 mm × 10 mm), were perpendicularly inserted into 1.2 inches. After the needling sensation was attained, the electrodes of G6805-2 electric stimulator were connected and electrostimulation was added. The intermittent wave was used, with a frequency of 20 Hz and an intensity that depended on the patients' tolerance. The electrical stimulation was given for 20 minutes once daily. (3) Treatment regimen: the total duration was 2 weeks, with 6 days therapy followed by 1 day off each week. (4) Other components of treatment: the intervention of the same WCM as the control group was also administered to the EA group. (5) Practitioner background: this entails an expert acupuncturist who has practiced acupuncture for more than ten years. (6) Control interventions: the patients at the control group were given WCM based on the *Chinese guideline for diagnosis and management of acute ischemic stroke *(version 2010) [14]. This stroke guideline is similar to the western countries [2], which means that management of patients with AIS remains multifaceted and includes several aspects of care that have not been tested in clinical trials: briefly, Bayaspirin 0.1 g (H20065051) q.d. (Clopidogrel 50 mg (H20000542) or Clopidogrel 75 mg (J20040006) q.d. if there is contraindication to aspirin treatment), Atorvastatin (Lipitor) 20 mg q.n., and combination of needed therapies of the following aspects for 14 days: (1) general supportive care mainly includes, (A) airway, ventilatory support, and supplemental oxygen, (B) cardiac monitoring and treatment, (C) temperature, (D) blood pressure, (E) blood sugar, and (F) nutrition; (2) specialized care mainly includes a variety of measures to improve cerebral blood circulation except thrombolytic agents and neuroprotective agents; (3) treatment of acute complications mainly includes, (A) brain edema and elevated intracranial pressure, (B) seizures, (C) dysphagia, (D) pneumonia, (E) voiding dysfunction and urinary tract infections, and (F) deep vein thrombosis. 

### 2.4. Outcome Assessments

The primary efficacy variables were TST, NIHSS, and FAM that utilized blind assessment at baseline and after 14 days of treatment. (1) TST: details of this technique are described previously by Magistris et al. [8, 10]. Briefly, the testing uses the Danish Dantec company's Keypoint.Net EMG and a MagPro Compact stimulator (Medtronic-Dantec, Skovlunde, Denmark) in the present study. TST consists of three stimuli, a first magnetic pulse to the motor cortex and a second and third supramaximal electrical pulse applied to a peripheral nerve, that is, the ulnar nerve over the wrist and over the plexus, respectively. The evoked antidromic and orthodromic responses collide in the nerve in a complex manner resulting in a compound muscle action potential (e.g., abductor digiti minimi muscle) providing a measure of the functional integrity of the corticospinal tract. A control trial without a TMS pulse and a test trial carried out as described above are compared. The TST amplitude ratio was computed using the formula TST_ratio_ = TST_test_/TST_control_. TST amplitude ratio and TST area ratio were used as a quantitative measurement of the central motor-conduction disorders. The ratio values indicated the percentage of central motor-conduction failure, and variation less than 10% was acceptable (TST_ratio_ should be >90% indicating that the corticospinal tract is functionally intact). In the present study, we used 1-TST_ratio_ as injury part of central motor-conduction impairment. (2) NIHSS: stroke outcome was assessed by NIHSS score [15]. (3) FMA: FMA is a stroke-specific, performance-based impairment index [16]. Poststroke hemiplegia patients were graded by FMA, in which the total score was 100 points and consisted of upper limb score 0–66 points and lower limb score 0–34 points.

The secondary efficacy variable was the clinical efficacy evaluation at the end of the treatment course by an appointed doctor and safety assessments. The criteria of neurological deficit score were adopted based on the Modified Edinburgh-Scandinavian Stroke Scale, a nationwide accepted scoring system recommended at the Fourth National Cerebrovascular Diseases Conference in China, including consciousness, gaze, facial paresis, language, walking ability, and motor function of arms, legs, and hands. The assessment was conducted in accordance with the reduction in the scores of basic nervous functional deficits and disability degree as follows: recovery scores: the functional deficit scores were decreased up to 91%–100%, and disability degree was at grade 0; remarkable improvement: the scores of functional deficit were decreased at 46%–90%, and disability degree was at the grade 1–3; improvement: the scores of functional deficit were decreased at 18%–45%; no change: the scores of functional deficit were decreased or increased at about 17%; deterioration: the scores of functional deficit were increased over 18%; and death [13]. 

### 2.5. Safety Assessments

Safety was assessed by the documentation of whole body reaction recorded whenever necessary, as well as by laboratory test of electrocardiogram (ECG), liver function, and kidney function both at baseline time and at the end time of treatment. 

### 2.6. Statistics Analysis

All data was processed by SPSS15.0. Quantitative data was described by mean ± standard deviation $\left(\overset{-}{x}\pm s\right)$. An independent sample *t*/*t*′-test was used for comparing the means between two groups; a paired *t*-test was applied for comparing the change in outcomes before and after treatment; Spearman's rho test was used for correlation analysis between the two quantitative variables (correlation coefficient, *r*). Qualitative data was described by the frequency (*f*) and the percentage (*P*). Fisher's Exact Test is a test for independence in a 2 × 2 table and Pearson chi-square test for R × C table. Statistical tests were completed blindly. All tests were two-sided and were considered to be statistically significant at *P* < 0.05.

## 3. Results

### 3.1. Baseline Data

Demographic characteristics and clinical features of participants in both groups are presented in Table 1. The differences in the demographics, including age, sex, comorbid disease, and infarct volume of the 2 groups, were insignificant (*P* > 0.05). Moreover, there is no statistically significant difference in all the preintervention-selected outcome measures such as TST, NIHSS, and FAM between EA group and WCM group (*P* > 0.05) (Table 1).

### 3.2. Clinical Effectiveness

 In the EA treatment group, there were 4 recovery cases, 20 remarkable improvement cases, 5 improvement cases, 1 no change case, and 1 deterioration case; the total effective rate was 93.50%. In WCM control group, there were 2 recovery cases, 14 remarkable improvement cases, 5 improvement cases, 8 no change case, and 1 worse case; the total effective rate was 73.33%. The total effective rate was statistically significantly superior in EA group to that in WCM group (*χ* ^2^ = 5.72, *P* < 0.01) (Table 2).

### 3.3. NIHSS Score, FMA Score, and TST_ratio_

**Figure 2:**
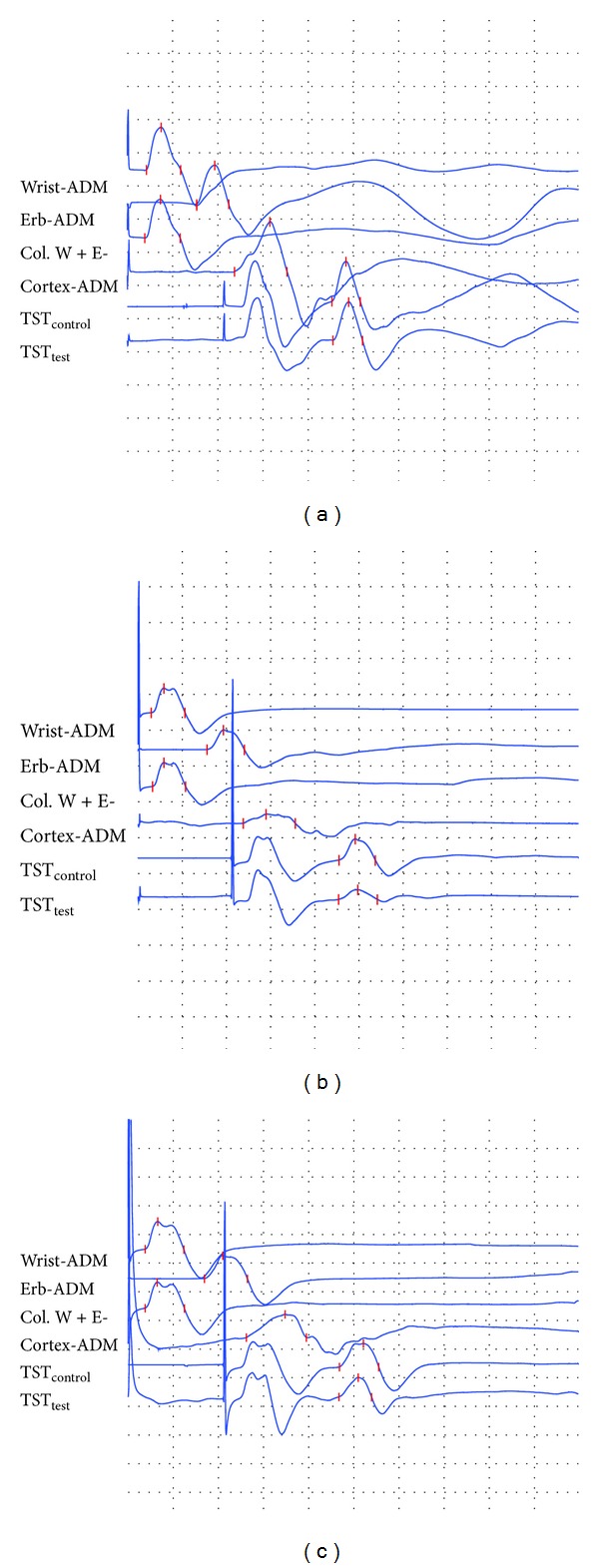
(a) Triple stimulation technique (TST) tested in the right ulnar nerve of an adult healthy subject. The TST amplitude ratio (TST_test_/TST_control_) was 90.1%. (b) TST tested in the right ulnar nerve of a patient with acute ischemic stroke. The TST amplitude ratio (TST_test_/TST_control_) was 45.8%. (c) TST tested in the right ulnar nerve of a patient with acute ischemic stroke after electroacupuncture treatment. The TST amplitude ratio (TST_test_/TST_control_) was 83.9%.

There was no statistical difference between EA treatment group and WCM control group in NIHSS score, FMA score, and TST_ratio_ before treatment (*P* > 0.05). After 14-day treatment, NIHSS score, FMA score, and TST_ratio_ between pretreatment and posttreatment were statistically more significant in EA treatment group than those in WCM control group (*P* < 0.01). In addition, there were significant differences between the two groups in all these three measure outcomes (*P* < 0.05) (Table 3, Figure 2).

### 3.4. Correlation of TST_ratio_ and NIHSS Score, FMA Score

 There was positive correlation between TST_ratio_ and NIHSS score before treatment (*r* = 0.646, *P* < 0.01) and after 14-day treatment (*r* = 0.649, *P* < 0.01). There was negative correlation between TST_ratio_ and FMA score both before treatment (*r*_*s*_ = −0.570, *P* < 0.01) and after treatment (*r* = −0.572, *P* < 0.01). There was also negative correlation between NIHSS and FMA scores both before treatment (*r* = −0.741, *P* < 0.01) and after treatment (*r* = −0.769, *P* < 0.01) (Table 4). 

### 3.5. Complications

No death of subjects or other serious adverse events occurred during the treatment period. Comparing between two groups or between pretreatment and posttreatment, adverse events, ECG, liver function, and kidney function showed no significant difference (*P* > 0.05) (Table 5). One patient in the EA group showed dizziness and limb weakness for 2 times after the TST test. The symptoms lasted for about half an hour and relieved by themselves. Alanine aminotransferase (ALT) or aspartate aminotransferase (AST) slightly elevated in three patients both in EA group and in WCM group, but the elevations were not greater than 2 times the upper limit of normal. Serum creatinine (SCr) level slightly elevated in two cases in WCM group (153 *μ*mol/L and 162 *μ*mol/L) and recovered after stopping use of the mannitol injection. Two cases had abnormal ECG with occasional atrial premature beats or the first degree atrioventricular block in EA group, and 1 patient had abnormal ECG with sinus arrhythmia in WCM group, all the above 3 patients with no requirement for clinical care. 

## 4. Discussion

 To our knowledge to date, this is the first randomized controlled study using the triple stimulation technique to objectively evaluate EA for motor function recovery in patients with AIS. The main findings in the present study were as follows: (1) EA had more beneficial effect on motor function recovery of AIS patients when compared with WCM control and was generally safe; (2) the effect of EA for motor function injury and recovery of corticospinal tract impairments in AIS patients can be quantitatively evaluated by TST. 

 Neurophysiological assessments have been used to measure the extent of stroke damage to the motor system and to predict subsequent recovery of function. An abnormal TST represents upper motor neuron loss, central axon lesions or conduction blocks, or inexcitability in response to TMS [17]. In healthy Chinese subjects, the TST amplitude ratio (TST_teat_/TST_control_) was 85.0% ± 6.7%, and there was no difference among genders, age groups, and arm length and sides [18]. In the present study, abnormal TST_ratio_ was observed in all included stroke patients, suggesting that TST is effective to assess corticospinal tract impairment in AIS. Therefore, TST can be a useful tool for quantitative diagnosis of corticospinal tract motor function in lesional defects of conduction after acute ischemic stroke.

Systematic reviews of the literature indicated that the initial grade of paresis as measured on admission in the hospital is the most important predictor of early prognosis of motor recovery, and special attention should be paid to the clinical prognostic value of MEPs [19, 20]. Severity of stroke measured by NIHSS score on admission is highly predictive of excellent or devastating outcomes in ischemic stroke patients [21]. FMA scale is a disease-specific objective impairment index designed specifically as an evaluative measure for assessment of recovery in the poststroke hemiplegic patient [16]. TST is a method improving the study of MEPs [8]. In the present study, TST and NIHSS score reduced, and the FMA score improved after treatment in both groups. TST was positively correlated with NIHSS scores and was negatively correlated with FMA scores after both EA and WCM treatment. These results showed that reduction of NIHSS scores and improvement in FMA scores indicated the improvement of motor function after treatment, and TST can be an indicator of prognosis for motor function recovery.

Along the clinical course of ischemic stroke, the most critical period of recovery is at the acute and subacute stages [12]. Although the evidence was limited due to the low methodological quality, at least 3 systematic reviews revealed the potential benefits of acupuncture to patients with acute ischemic stroke [4, 22, 23]. In the present pilot study, EA is more effective in patients with first-ever AIS for motor function recovery when compared with a WCM control using NIHSS score for the neurologic severity assessment and FMA score for the motor-status evaluation. This result was compatible with the several previous studies [24–26]. Particularly, we used TST to objectively quantify assessment of the central conduction failure after EA treatment. Comparing the WCM control, EA treatment during the acute phase of stroke can significantly improve corticospinal conduction failure, suggesting that EA had additional beneficial effects on motor functional outcome and TST can be an accurate and objective quantification in assessment of the efficacy of EA treatments.

 Acupuncture appears to be a safe treatment when used in the acute phase of stroke, with rare serious adverse events [22]. An NIH consensus report also stated that one of the advantages of acupuncture was that the incidence of adverse effects is substantially lower than that of many other accepted medical interventions [27]. Although EA is a somewhat invasive procedure with complications such as needle pain, fainting, minor bleeding, or infection, few side effects were noted in the present study. Thus, the present study supported the safety of EA for AIS patients.

 A few comments about the design methods and study limitations deserve mention. First, one potential limitation of this pilot study is the small sample size evaluating the efficacy and safety of EA for AIS. Trials with inadequate sample sizes could run the risk of overestimating intervention benefits [28]. Another limitation is that the follow-up data after treatment were not available. Lack of followups led to difficulty in accounting for the long-term efficacy of EA treatment for stroke. Finally, a true double-blinded acupuncture trial would be very difficult to carry out because the acupuncturist always knows which method is being applied, and the patient can easily distinguish between active and nonactive stimulation. Although some placebo/sham acupuncture methods have been invented in the past decade [5], the use of these methods in control groups remains controversial. Therefore, it cannot be guaranteed that the placebo effect of the EA treatment had been removed to some extent from the results, even though WCM group was taken as control in the present study. 

In conclusion, EA had more beneficial effect on motor function recovery of AIS patients after 14-day treatment when compared with WCM control and was generally safe. TST can quantitatively evaluate EA for motor function recovery in patients with AIS by objective analysis of the injury and recovery of corticospinal tract impairment. Further rigorously designed large sample size randomized double blind clinical trials are required. 

## Figures and Tables

**Table 1:** Baseline of demographic and clinical characteristics.

Variables	EA group (*n* = 31)	WCM group (*n* = 30)
Age (years)	57.35 ± 12.83	60.30 ± 12.16
Sex		
Male	15	21
Female	16	9
Comorbid disease		
Hypertension	19	24
Diabetes mellitus	3	6
Coronary heart disease	2	5
Infarct volume		
Large size	4	1
Medium size	6	9
Small size	21	20
NIHSS score	6.10 ± 2.61	6.30 ± 3.10
FMA score	68.48 ± 19.81	65.30 ± 26.61
TST_ratio_ (%)	63.54 ± 28.20	64.77 ± 26.80

EA: electroacupuncture; FMA: Fugl-Meyer Assessment Scale; NIHSS: National Institutes of Health Stroke Scale; TST: triple-stimulation technique; WCM: western conventional medication.

**Table 2:** Comparison of the clinical efficacy between EA treatment group and WCM control group (*n* (%)).

Group	*n*	Recovery	Remarkable improvement	Improvement	No change	Deterioration	Death	Total effective rate^#^
EA	31	4 (12.9)	20 (64.5)	5 (16.2)	1 (3.2)	1 (3.2)	0 (0.0)	93.50%*
WCM	30	2 (6.7)	14 (46.7)	5 (16.7)	8 (23.3)	1 (3.3)	0 (0.0)	73.33%

EA: electroacupuncture; WCM: western conventional medication. Compared with WCM control group, *P < 0.05. ^#^Total effective rate = (recovery + Remarkable improvement + improvement) × 100% ÷ *n*.

**Table 3:** Comparison of NIHSS score, FMA score and TST_ratio_ between EA treatment group, and WCM control group $\left(\overset{-}{x}\pm s\right)$.

Items	Group	*n*	Assessment time	
			Pretreatment	Posttreatment
NIHSS score	EA	31	6.10 ± 2.61	2.52 ± 1.93^∗##^
	WCM	30	6.30 ± 3.10	4.17 ± 3.40^##^
FMA score	EA	31	68.48 ± 19.81	85.27 ± 16.59^∗##^
	WCM	30	65.30 ± 26.61	74.17 ± 24.16^##^
TST_ratio_ (%)	EA	31	63.54 ±28.20^#^	39.91 ± 31.76^∗##^
	WCM	30	64.77 ± 26.80	56.45 ± 31.49^##^

EA: electroacupuncture; FMA: Fugl-Meyer assessment scale; NIHSS: National Institutes of Health Stroke Scale; TST: triple-stimulation technique; WCM: western conventional medication. Compared with WCM control group, *P < 0.05. Compared with pretreatment, ^#^ *P* < 0.01.

**Table 4:** Correlation of TST_ratio _and NIHSS score, FMA score (*r*).

	Pretreatment			Posttreatment		
	NIHSS	FMA	TST	NIHSS	FMA	TST
NIHSS	1			1		
FMA	−0.741**	1		−0.769**	1	
TST	0.646**	−0.570**	1	0.649**	−0.572**	1

FMA: Fugl-Meyer assessment scale; NIHSS: National Institutes of Health Stroke Scale; TST: triple-stimulation technique. Correlation, **P < 0.01.

**Table 5:** Comparison of adverse events, electrocardiogram, liver function, and kidney function between two groups or between pretreatment and posttreatment.

Group	*n*	Pretreatment		Posttreatment			
		ALT	SCr	Adverse events*	ECG*	ALT	SCr
EA	31	23.43 ± 5.06	73.61 ± 15.07	1	2	24.08 ± 4.65	74.38 ± 14.77
WCM	30	23.32 ± 4.48	73.61 ± 16.01	0	1	25.12 ± 5.88	73.82 ± 13.88

ALT: alanine aminotransferase; EA: electroacupuncture; ECG: electrocardiogram; SCr: serum creatinine; WCM: western conventional medication. *One patient at 2 times in the EA group showed dizziness and limb weakness after the TST test which lasted about half an hour and relieved itself. **Two cases had abnormal ECG with occasional atrial premature beats or the first degree atrioventricular block in EA group, and 1 patient had abnormal ECG with sinus arrhythmia in WCM group, all with no requirement for clinical care.
